# Supplementary material for: Convolutional neural network using magnetic resonance brain imaging to predict outcome from tuberculosis meningitis
Source: PLoS One. 2025 May 23;20(5):e0321655. doi: 10.1371/journal.pone.0321655 (PMC12101703; doi:10.1371/journal.pone.0321655)
Supplement: S1 Fig — Each model was trained on 4 in 5 folds in the whole training set and was used to predict the one held-out fold. All predictions and ground truths on these held-out folds were subsequently concatenated to recover the full training set (herein denoted as pooled validation set) to calculate AUC and CE loss. The hyperparameter set that maximized AUC and minimized CE loss were selected [file pone.0321655.s001.pdf]

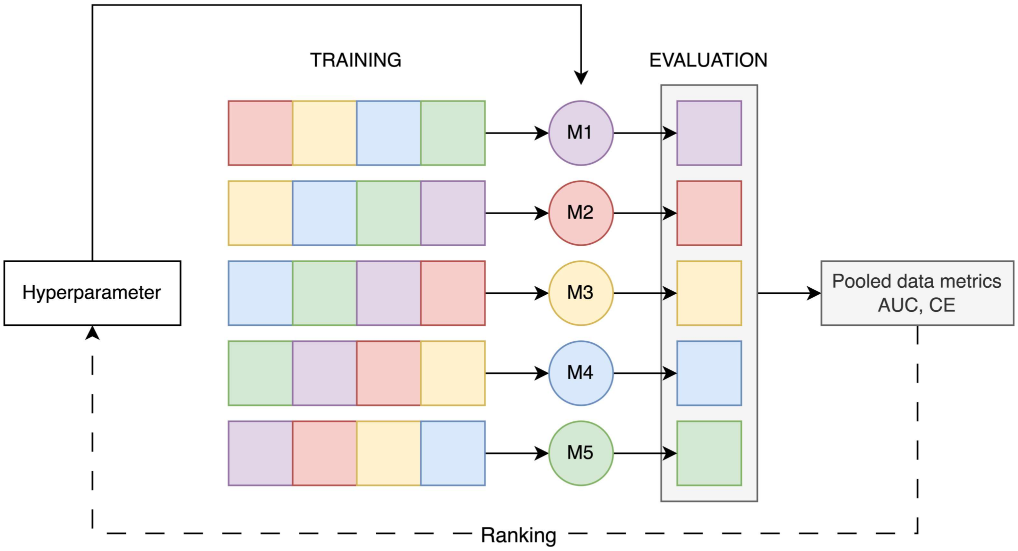

Supplementary Fig S1. Hyperparameter grid search and evaluation strategy. Each model was trained on 4 in 5 folds in the whole training set and was used to predict the one held-out fold. All predictions and ground truths on these held-out folds were subsequently concatenated to recover the full training set (herein denoted as pooled validation set) to calculate AUC and CE loss. The hyperparameter set that maximised AUC and minimised CE loss were selected
