# Supplementary material for: Convolutional neural network using magnetic resonance brain imaging to predict outcome from tuberculosis meningitis
Source: PLoS One. 2025 May 23;20(5):e0321655. doi: 10.1371/journal.pone.0321655 (PMC12101703; doi:10.1371/journal.pone.0321655)
Supplement: S1 Table — Values that minimized the validation loss were selected into the final model. [file pone.0321655.s002.pdf]

Supplementary Table S1. Definition of hyper-parameters that were grid-searched by cross-validations. Values that minimised the validation loss were selected into the final model.

Term	Definition
LR initial value	The update size in each optimisation step
LR scheduler	After k optimisation iterations/epochs, LR in multiplied by γ, i and γ are hyper-parameters that were searched. Hence at epoch $i > 0$: $$\lambda(i) := \lambda(0) \times \gamma^{\lfloor \frac{i}{k} \rfloor}$$
Dropout probability	In each training epoch, this term governs how many percentages of neurons in one layer are set to 0.
Weight decay	After each training epoch, the weights of each neuron are multiplied by the term accordingly. This is akin to Ridge penalty term in $\mathrm{M}_{\mathrm{clin}}$
