# Supplementary material for: Convolutional neural network using magnetic resonance brain imaging to predict outcome from tuberculosis meningitis
Source: PLoS One. 2025 May 23;20(5):e0321655. doi: 10.1371/journal.pone.0321655 (PMC12101703; doi:10.1371/journal.pone.0321655)
Supplement: S2 Table — with the results of the grid search reported on column “Value.” [file pone.0321655.s003.pdf]

Supplementary Table S2. Hyper-parameters for image augmentation that were searched, with the results of the grid search reported on column "Value"

**Transformation**	**Hyper-parameters**	**Search space**	**Value**
Random flipping	Probability of applying the transformation	[0.5]	0.5
Random affine	Probability of applying the transformation	[0.25, 1]	1
	Rotate range (rad)	[$\pm\pi/36$, $\pm\pi/4$]	$\pm\pi/18$
	Translate range (px)	[5, 20]	15
	Shear range (px)	[±0.1, ±0.3]	±0.1
	Scale range (-1, 1)	[-0.2, +0.2]	±0.1
Random intensity scale	Probability of applying the transformation	[0.1, 0.75]	0.75
	Scale factor	1 ± [0.1, 0.3]	1 ± 0.2
Gaussian noise	Probability of applying the transformation	[0.25, 1]	0.5
	mean of noise	0	0
	standard deviation (sd) of noise	[0.01, 0.05]	0.02
Gibbs noise	Probability of applying the transformation	[0.25, 1]	0.5
	min and max of noise intensity	[0, 0.2]	(0, 0.1)
Coarse dropout	Probability of applying the transformation	[0.25, 1]	0.75
	min and max size of dropped out voxels	[2, 20]	(5, 20)
	number of patches	[10]	10
