# Supplementary material for: Convolutional neural network using magnetic resonance brain imaging to predict outcome from tuberculosis meningitis
Source: PLoS One. 2025 May 23;20(5):e0321655. doi: 10.1371/journal.pone.0321655 (PMC12101703; doi:10.1371/journal.pone.0321655)
Supplement: Attachment — Submitted filename: Response to Reviewers.pdf [file pone.0321655.s004.pdf]

Convolutional neural network using magnetic resonance brain imaging to predict outcome from tuberculosis meningitis

Response to reviewers

Submission ID PONE-D-24-44674

Corresponding author: Trinh Huu Khanh Dong

Overall: We would love to express our appreciation to both reviewers for your kind and detailed comments to improve the quality of our manuscript. We acknowledge that there are some issues with the content, as pointed out by the reviewers. We, hence, have carefully taken care of your points of correction and made replies and corrections on our manuscripts, where appropriate. Please kindly find below our point-by-point responses and the related lines in the **resubmitted manuscript with tracked changes**, as requested by the journal. To produce the tracked change version, we used package `latexdiff` as recommended. Removed text is marked with red colour and strike-through, while added text is marked in blue. Due to the complexity of the table structure, changes made within table cells were not marked automatically. We have highlighted in blue the changes manually.

We have made point-by-point responses to each reviewer, in two separated tables below. In each table, the original question is on the left-hand column, coupled with our response on the middle. On the rightmost column are the relevant line number (if any) in the **change-tracked version** that contain the change we made in accordance with the point of correction, for easier lookup. We hope that out responses will be satisfying to the Reviewers. Our deepest apologies to the reviewers for any inconvenience with the aesthetics of the change-tracked file. Please kindly refer to the **untracked LaTeX/PDF version** for the true intended visual appearance and structure.

Reviewer 1:

Points of corrections	Response	Revised lines (tracked changes)
Accessibility for Clinicians: The manuscript is highly technical, which may limit its practical application for clinicians in routine care.<br>Simplifying the language and providing more direct clinical insights would enhance its relevance and usability in daily practice.	Thank you for your considerate comments. As a clinician by training, I admit that the technical level of this manuscript is quite high, which hampers the accessibility for the clinicals. During the time of writing, we have attempted to balance the technical transparency and the medical impact. However, there are some technical aspects that we cannot sacrifice for the transparency of the methodologies, which also an aspect to determine the credibility of the results.<br>However, we agree with the Reviewer that, we need to add some additional medical input to target the clinicians in the field. We have hence added some medical points in the Discussion part, which also associates with your third points.	413-477
Definition of Outcomes: The definition of "events" as outcomes in this paper seems somewhat arbitrary. Death and neurological deficits represent distinct outcomes with a wide range of severity. It would be beneficial for the authors to provide a clear justification for grouping these outcomes together or, alternatively, analyze them separately.	Thank you for your concern. The definition of the events in general followed the definition of primary endpoints in the two main RCTs that provided the dataset (ACT HIV and LAST ACT) [20, 21]. In particular, the protocol of LAST ACT stated that "The primary endpoint is death or new neurological event (defined as a fall in Glasgow coma score (GCS) by ≥2 points for ≥2 days from the highest previously recorded GCS (including baseline) or the onset of any of the following clinical adverse events: cerebellar symptoms, focal neurological signs, or new seizures) during 12 months from randomisation."<br>From a clinical perspective, as neurological complications ignited by TBM are, more likely than not, irreversible. Survivors with neurological events during treatment course tend to suffer long-term cognitive impairment and limit in their activities afterwards. The combination of both outcomes highlights that they are both clinically meaningful as a TBM outcome.	We have added some text to clarify our choice of outcome: line 155-157

Points of corrections	Response	Revised lines (tracked changes)
	From a modelling perspective, separating the two outcomes would complicate the model, as the two outcomes can be overlapped (those who had neurological events could die consequently), diluting the main objective without much gain in the clinical impact.	
MRI Findings and Model Explanation: The discussion of the MRI findings and the defined model is insufficiently detailed. The authors should expand on how specific MRI lesions relate to clinical features and outcomes, providing more thorough explanations and discussions to strengthen the paper's conclusions.	We thank the Reviewer for pointing this out. As the MRI finding, in particular the saliency maps, are purely post hoc analysis without uncertainty estimation, we were extremely careful not to over-interpret the results. We acknowledge that it may be more accessible to the clinician with more clinical input. We therefore added some details regarding our observations from the post hoc analysis and provided some additional details on the corresponding figures.<br><br>However, we also highlight that, these findings are purely observation and not rigorously checked. To overcome this limitation, we also had another paper in plan which leverages brain parcellation to fully segmentate the brain regions. By having a clear definition of brain regions, we can make the interpretation more credible and accessible. This uses a completely different method and will come in a later paper.	357-376<br>379-381<br>413-477<br>510-514<br>Fig. 6 (above 372)

Reviewer 2:

Points of corrections	Response	Revised lines (tracked changes)
The study of Canas et al. (ref 40) is using the same dataset and it should be mentioned in the beginning what the difference is with this study since there is clearly some overlap. In the discussion one cannot use this study to discuss similarities since this is expected if you use similar methodology on the same dataset. This part of the discussion should be removed.	I would like to express my thank for the Reviewer's comment. Both manuscripts were two parts of a three-part analysis where we performed different investigation on imaging biomarkers for TBM. The datasets indeed shared some overlap, but the methodologies and the outcome were different. In this manuscript, we investigated the short-term outcome during treatment course (6 week) of dexamethasone using only baseline MRI while Liane et.al. used time series MRI to predict long-term quality of life after discharge. Initially, we thought it was of interest to share some similarity in findings using different methods. However, I admitted that the Reviewer's concern is very sensible and understandable. We hence decided to remove the related paragraph from the Discussion.	459-467
Performance values were compared between the HIV positive and HIV negative group but this is only descriptive and no statistical analysis is performed. Without a proper statistical analysis, we cannot conclude much from a descriptive observation.	As the sample for the subgroup analysis was small, we constrained ourselves from performing too many statistical testing, because (1) multiplicity correction may bear the risk of nullifying our findings and (2) we did not how to perform correction in this particular case, as the tests were not independent. A potential method is to "pretend" that this was a sequential setup (HIV+ and then HIV-) and performed a Spiessens-Debois correction (https://pubmed.ncbi.nlm.nih.gov/20832503/) which exploits the correlation between them to save some error type I. However, this is (1) complicated and (2) not guaranteed to be fully correct.<br>We agreed with the Reviewer. Your concern is absolutely valid. We hence therefore added to the Discussion, stating that this comparison is purely speculative.	340<br>403-406

Points of corrections	Response	Revised lines (tracked changes)
Figure 1: The arrow in panel C is not very well visible and this can be improved.	Thank you for your comment. We have made the arrow bigger. Please also kindly note that I have noticed that the quality of the figures sent by the journal is not ideal. The original figures had at least 300 dpi – usually 600 dpi.	Fig 1 (above 66)
Who checked the quality of the MRI? Was it visually checked by a neuroradiologist?	We performed the visual check on the correctness of sequence, artefact (namely Gibbs noise, contrast, bias field, or any irrecoverable deformation) and field of view of the scans (missing slices, invasive crop). We did not check for any lesion appearance as it might bias the analysis. We have TBM clinicians and medical image engineers in the team who had experience with MRI.	122
How many scans were excluded?	The total number of excluded sessions were 49, amongst which 13 were due to QC failure. The numbers were explicitly mentioned in Fig 4 and on line 275.<br><br>Scan-wise, there were in total 56 T1W scans excluded from the analysis (36 HIV-negative, 20 HIV-positive). In most of the cases, the radiographers would have noticed that the scan were unusable and performed an alternative scan in the same session, explaining the fact that there were more scans excluded than sessions. As there were usually alternative usable scans, we believe that the number of excluded scans was not relevant to the analysis.	276
The authors mention that a patient was excluded if no other scan was available as a replacement but this is unclear. Did you included other sequences besides a T1w-MPRAGE or did some patients have multiple scans? If the latter	My apologies we did not make this point clear. We only curated T1w-MPRAGE sequence. If the participants had multiple T1w-MPRAGE sequence, we got the first session, not later than one month (as advised by the TBM experts) nearest to the enrolment date. If there were many T1w-MPRAGE scans in the first session, we got the scan with best quality by manual QC.	124-128

Points of corrections	Response	Revised lines (tracked changes)
is true, which image was used or were all images used?	Any T1w-MPRAGE scan that did not pass the manual QC would be excluded. If all T1w-MPRAGE was removed (due to this reason), the patient was excluded. If the patient died before any MRI was taken, they weren't included in the analysis.	
Missing variables were imputed within the training process and this is a valid technique provided that the number of missing data is small compared the collected data. How many missing data were present for the different variables?	In Table 3 (Baseline Characteristics), column N represented the number of observed data points for that particular variable. An N < 215 would indicate missing data. We apologies that was an implicit representation and we apology for any confusion this may have made. We hence explicitly showed the number of missing points in a dedicated row in Table 3, denoted as "(Missing)", representing the number of missing data points for the variables preceding it, stratified by HIV status.	Table 3 (above line 288)
MRI scans were rigidly registered into MNI space but I assume that scaling was applied as well. This could be mentioned explicitly.	We sorry for the confusion and have changed the wording to "affine-registered".	141
Intensities were normalized to range [0,1]? How was this done exactly? Scaling with respect to the maximum in the image can be a problem depending where this voxel is located.	Thank you. That is a great question. We believe that we need to scale the MRIs into some comparable intensity scale. However, choosing which is still a question under debate. We opted in a trial-error strategy by experimenting with both scaling by range and by standard deviation (sd) before feeding into the neural network.<br>For some unknown reason, we found that the model training was more likely to fail with sd-scaled images (stuck and did not move) When it did not fail, we found effectively no difference in performance compared with the range-scaled images. To ensure that the transformation did not corrupt the image, we also	146-147

<table>
<tr><th>Points of corrections</th><th>Response</th><th>Revised lines (tracked changes)</th></tr>
<tr><td></td><td>visually checked the scaled and registered images for validity before inputting them into the model. We have clarified that in the text.
In conclusion, we performed the following scaling, where x_i is the intensity at voxel i:
$$T(x_i) = \frac{x_i}{\max(x_i)}$$</td><td></td></tr>
<tr><td>It would be interesting to see a correlation matrix between all the non-imaging features. How are features which are highly correlated (if present) handled?</td><td>This is the correlation between non-imaging continuous features (age, weight, GCS, and CSF lymphocyte count). There are some weak correlations between variables, as shown below.
<table>
<tr><th></th><th>age</th><th>weight</th><th>gcs</th><th>csf_lym</th></tr>
<tr><td>age</td><td>1.00</td><td>0.12</td><td>-0.10</td><td>0.09</td></tr>
<tr><td>weight</td><td>0.12</td><td>1.00</td><td>0.00</td><td>0.15</td></tr>
<tr><td>gcs</td><td>-0.10</td><td>0.00</td><td>1.00</td><td>0.15</td></tr>
<tr><td>csf_lym</td><td>0.09</td><td>0.15</td><td>0.15</td><td>1.00</td></tr>
</table>
While multicollinearity may interfere the association between the outcome and one particular feature, we expect them to have minimal impact on predictive value of the model (as explained here https://stats.stackexchange.com/questions/361247/multicollinearity-and-predictive-performance). As we did not perform inference on the non-imaging features, we did not perform any correction for multicollinearity.
As for the inference on the imaging feature, we used SmoothGrad, which is a non-linear technique based on the gradient and not the coefficients. As the gradient was calculated altogether for all feature point (both imaging and non-imaging), any collinearity, in theory, should have been taken into account. However, we are not sure if in practice that statement is true, as it needs further research.</td><td></td></tr>
</table>

Points of corrections	Response	Revised lines (tracked changes)
The caption of figure 2 is very minimal and should be extended.	We thank the Reviewer for the great comment. We have extended the figure captions.	Fig 2 (above line 174)
It is unclear to me how the vector of 512 imaging features is compressed into a latent vector of length 24.	This is a layer of a feed-forward neural network, effective a matrix multiplication between a matrix whose dimension = 24 x 512 (parameters) and a vector of length 512 (features). Visually,<br>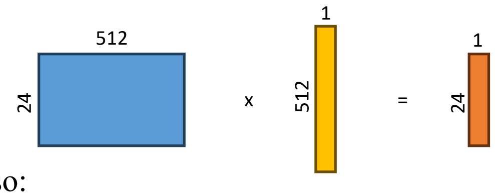<br><br>See also:<br>https://en.wikipedia.org/wiki/Matrix_multiplication<br>We also made add a vector of 512 into Fig 2 to make this layer more clearly.	Fig 2<br>(above line 174)
P7, line 206: it is mentioned that equation 2 (with alpha = beta = 1) is analogous to concurrently maximizing the likelihood of the three tasks but I am not sure if this is indeed the case. I would expect that this depends on the relative loss with respect to the others.	That is a valid point. We are sorry for the confusion. The idea is, as Cross Entropy loss is effective the Negative Log Likelihood of a binomial regression (https://en.wikipedia.org/wiki/Cross-entropy#Relation_to_maximum_likelihood), summing up the Cross Entropy loss would, in theory, summing up the Negative Log Likelihood.<br>As log(x)+log(y) = log(xy), we effective have the log of multiplication of the likelihood.<br>Minimising the total Cross Entropy loss would maximise the muliplication of likelihood. If the three tasks are independent, this is translatable to maximising the likelihood function of each tasks, as<br>P(A) x P(B) = P(AB) given A&B are iid.<br>However, we noticed that the three tasks are not mutual independent (only M_{img} and M_{clin} are independent), the formula did not translate well to our statement. Therefore, we decided to remove the confusing statement.	214-215

Points of corrections	Response	Revised lines (tracked changes)
	The formulation/treatment is still useful to constrain the model to balancely optimise the two auxilary tasks L_{clin} and L_{img} while still make them contribute to the other (by optimising the L_{fused}).<br>However, this formulation comes with a trade-off: the loss of calibration to the large, which is a characteristics of binomial regression when likelihood is maximised (https://stats.stackexchange.com/questions/390487/why-is-logistic-regression-well-calibrated-and-how-to-ruin-its-calibration). We denote this a improper loss function. This is linked to another question of yours that we will discuss below.	
P7, line 232: the model was recalibrated by fitting an intercept-only logistic regression on the observed class and the predicted scores as the offset in order to correct for class imbalance. Is there a reference for this procedure showing that this will indeed correct for class imbalance?	Thank you very much for pointing this out, this is a typing mistake from us. It should be "to correct for improper loss function". We have made the change accordingly.<br>As discussed above, combining the losses makes the model miscalibrated due to a bias loss. This may hold true (but not as severe) with Ridge loss in penalised logistic regression. The level of miscalibration depends on the penalty term value, which differs by models. This miscalibration does not affect ranking-based performance metrics (like AUROC, AUPRC, balanced accurary) but severely impact probability-based metrics (like calibration curve, Brier score, Scale Brier Score, or Net Benefit). Therefore, when comparing model performance by Scale Brier Score, we had to recalibrate them, at least to the large (by using validation set in each cross-validation) to make models comparable. The choice of using event prevalence as a decision threshold (to calibrate) was inspired by Van Calster et.al (https://bmcmedicine.biomedcentral.com/articles/10.1186/s12916-019-1466-7,	240

Points of corrections	Response	Revised lines (tracked changes)
	https://academic.oup.com/jamia/article/29/9/1525/6605096).	
It is not clear what the smooth gradient based saliency maps are representing? Is it some sort of relative weight of a voxel which reflects its contribution to the final output?	You are correct. In brief, the backbone of SmoothGrad used back propagation and calculate the gradient of each voxel given the true class (in this case, "event" – death of any neurological complication). The result is an image of gradient, visualised in the form of a heatmap/salicency map. **The larger the gradient, the more contribution the corresponding voxel/feature is.** So, it is indeed possible to interpret the saliency map as some relative weights of a voxel reflecting its contribution to the final output.<br><br>The problem of this gradient-based method is that they can be noisy – demonstrating some uncertainty of the model.<br>The "Smooth" extension hence utilies an addition of random Gaussian noise onto the input images, then recalculate the gradient for each noisy images. By doing that numerous times and averaging out all the resulted maps, we acquire a more stable version of the saliency map compared to backbone-based saliency map above.<br>More information can be found in the original paper: https://arxiv.org/abs/1706.03825<br>We have added some clarification text in the Method Section.	267-269<br>357-372<br>Fig 6 (above 372)
In table 3, event should be replaced by new neurological event (including death) within 2 months or is it representing something else?	Event is indeed death or any neurological events within 2 months (or 60 days). We apology for not making this clear. We have clarified the definition in the footnote of the Table.	Table 3 (above line 288)

Points of corrections	Response	Revised lines (tracked changes)
The age of 24 (1st quartile for the whole group) is different form the value in the text which is 25.	Thank you very much for pointing this out. We wrote the calculation in R but did not notice that there were some difference in calcuating quantils between packages. This discrepancy is due to the different methods used to estimate quantiles for ties in the Table 3 and in the text. We have fixed the function to make them consistent. Thank you for pointing this out.	281
I would also add a separate table for the test set to see how representative this test set is.	Thank you very much for your suggestion. That makes perfect sense. We have added the table of interest, denoted as Table 4, stratified by data splitting.	Table 4 (below line 298)<br>299-301
P10, line 271 it is mentioned that 79 out of 215 participants (50.2%) were infected with HIV but I don't understand what the 50.2% is representing. 79 out of 215 is much smaller than 50%.	Thank you very much for your suggestion. We are sorry for this coding error. The number in the parenthesis should be 36.7%	285
Is there a reference or an argument why the authors selected some of the hyperparameters such as weight decay at 0.12 for each feature?	We apology for the confusing word. They were not "chosen" but rather "searched" by 2 repeated 5-fold cross validation. The number that minimised the validated Cross Entropy loss would be selected in the final model. The same goes for transformation parameters in Table 5.	306-309<br>Table 5 (above line 329)
Figure 5: the quality of the figure is poor and values and text were difficult to read. I would also recommend to use the same scale for panels C and D and for panels E and F so that the reader can more easily compare the	We are pretty much sorry for the quality of the figures. From the download after submission, we did notice that the quality of the figures exported by the journal is not ideal. In our original manuscript, all figures had at least 300 dpi (dots per inch). We do not know how to fix this with the journal. In the revision, I have uploaded a zip file, named Figures_Original_Resolution. Please kindly download it for original resolution.	Fig 5 (above line 338)

Points of corrections	Response	Revised lines (tracked changes)
results of the validation and test set. The n=10 is confusing. I would rather mention the actual number of patients used.	The n=10 is the number of fold-based models (2 times x 5 folds) that we fitted. As outlined in Fig 3, each model was tested on 1/5 of each cross-validation split, the performance metrics were then informally compared in a paired statistical test.<br>Thank you very much for the suggestion about the plots. That is a brilliant idea which could help a lot in readability. We have made the scale on C-D, and E-F consistent.	
In figure 6, I do not see coronal planes while this is mentioned in the caption. I would also add a colour bar.	You are absolutely correct that there were no coronal planes. That was a typo from us. We hence removed the text accordingly. We also added the colour bar as requested.	Fig 6 (above line 372)
