# Supplementary material for: Convolutional neural network using magnetic resonance brain imaging to predict outcome from tuberculosis meningitis
Source: PLoS One. 2025 May 23;20(5):e0321655. doi: 10.1371/journal.pone.0321655 (PMC12101703; doi:10.1371/journal.pone.0321655)
Supplement: Attachment — Submitted filename: Response_to_Reviewers_auresp_2.pdf [file pone.0321655.s005.pdf]

Convolutional neural network using magnetic resonance brain imaging to predict outcome from tuberculosis meningitis

Response to reviewers

Submission ID PONE-D-24-44674

Corresponding author: Trinh Huu Khanh Dong

Dear Reviewer,

We appreciate the Reviewer's comment and agree that the previous version of the manuscript placed a significant emphasis on the method, mainly for transparency and reproducibility purposes. In summary, the Reviewer made two points: the lack of a tracked-changes version (point 1) and the overly focus on methodology (point 2). We will first focus on point 2, which is the primary concern of the Reviewer.

For point 2, we agree that this overfocus on methodology might have given the manuscript a less attractive position to clinical readers. Please kindly note that while our proposed approach and full framework are novel in their application to the present clinical problem of *tuberculous meningitis*, the core model itself is not entirely new and has been applied to other diseases. To address that point, and by aiming to refocus the paper on its clinical contribution, we have moved details regarding the method to supplementary material, from both the Methods and Results sections as follows:

- **Table 2** moves to the Supplementary document and becomes **Supplementary Table S1**.
- **Table 5** moves to the Supplementary document and becomes **Supplementary Table S2**.
- **Figure 3** moves to the Supplementary document and becomes **Supplementary Figure S1**.

Furthermore, to emphasise the clinical value of our findings, we have modified the Discussion to describe better the clinical conclusions aroused from our results, as kindly suggested by the Reviewer, as follows:

- In the first paragraph, we have reiterated the clinical context and challenges that motivated our study.
- In the second paragraph, we have restated our clinical objective and introduced the main clinical results for the objective.
- In the third to eighth paragraphs, we discussed the results in detail.
- In the ninth to fourteenth paragraphs, we discussed our pros and cons.
- In the last paragraph, we reiterated our study and clinical impact.

We hope this addresses the Reviewer's main concerns and makes our study message clearer to the journal audience.

For point 1 made by Reviewer regarding the absence of a tracked-change file, please kindly accept our deepest apologies. We have contacted the journal's editor. However, they did not find any issues. Please kindly find a PDF document with the name "**Revised Manuscript with Track Changes.pdf**". If you are

receiving a long PDF file with all the forms, manuscript, and download links to supplementary documents embedded, please kindly scroll down to the near bottom. I would assume you can find the tracked-changes version there, followed by a PDF version of this Response letter. For some strange reason, I am not allowed to reorder the documents when I uploaded. In the case it does not appear on your side, please kindly contact the editor or the journal, since the existence of this file is mandated by them during the resubmission process. For the resolution of the figures, please kindly find in the attached file **Figure.zip** where Figures are sent in their original resolution. The downscaling of the figures was not intentional. Please kindly refer to the **untracked LaTeX/PDF version** for the true intended visual appearance and structure.

With best regards,
